# Multifaceted challenges of deep venous thrombosis in the setting tetraplegia and ulcerative colitis: case report

**DOI:** 10.1038/s41394-025-00703-3

**Published:** 2025-04-01

**Authors:** Priscilla Mapelli, Mitchel Wright, Henry Hrdlicka, David Rosenblum

**Affiliations:** 1 University of Connecticut School of Medicine, Physical Medicine & Rehabilitation Residency Program, Farmington, CT USA; 2 Gaylord Specialty Healthcare, Department of Physical Medicine & Rehabilitation, Wallingford, CT USA; 3 Gaylord Specialty Healthcare, Milne Institute for Healthcare Innovation, Wallingford, CT USA

**Keywords:** Neurophysiology, Diagnosis, Ulcerative colitis

## Abstract

**Introduction:**

Traumatic spinal cord injury (SCI) tetraplegics are at an increased risk of deep venous thrombosis (DVT) due to immobility and altered hemostasis. Inflammatory bowel diseases such as ulcerative colitis (UC) face an elevated risk of thrombotic events due to chronic inflammation, in addition to the risk of diarrhea and bleeding. The case report underscores the potentially additive prothrombotic effects of ulcerative colitis and tetraplegia.

**Case presentation:**

A 53-year-old male with UC and traumatic R C3 L C4 sensory, R C3 L C5 motor ASIA impairment C tetraplegia, developed a below the knee DVT during inpatient rehabilitation, despite DVT prophylaxis. Due to potential risk of progression, interventions ultimately included serial ultrasound examinations, IVC filter, and anticoagulation. However, due to bleeding complications, anticoagulation was discontinued, followed by worsening of DVT to the bilateral lower extremities which advanced above the knees. Subsequently, the patient developed clostridium difficile infection, further exacerbating his ulcerative colitis. Bowel program was impacted, and treatment was provided for both clostridium difficile and ulcerative colitis.

**Discussion:**

Both UC and traumatic SCI increase have risk of thrombosis. UC exacerbations and bleeding pose challenges in the treatment of DVT. The need to discontinue anticoagulation due to bleeding risk led to a significant progression of the DVT. SCI bowel program required careful adjustments in the setting of an UC exacerbation, likely triggered by clostridium difficile infection.

## Introduction

Patients with traumatic spinal cord injury (SCI) tetraplegia have an increased risk of deep venous thrombosis (DVT) due to immobility and altered hemostasis. Loss of typical sympathetic tone throughout the body may lead to changes in the spinal cord-gut-immune axis, causing cardiovascular, metabolic, and neurological complications, exacerbating co-morbidities that were present before SCI [1].

Inflammatory bowel diseases (IBD), such as ulcerative colitis (UC), can also increase the risk of venous thromboembolism (VTE) due to chronic inflammation, diarrhea, and gastrointestinal (GI) bleeding. The pathogenesis of UC hypercoagulability is poorly understood; however, it is likely due to the upregulation of pro-coagulants, impairment of the fibrinolytic system, and lower levels of natural anticoagulants [2]. In this case, the patient presented with both acute traumatic tetraplegia and a history of chronic UC, which significantly increased the risk of VTE.

## Case presentation

In August 2023, a 53-year-old male with a significant past medical history of chronic UC presented to the Gaylord Hospital (Wallingford, CT) inpatient rehabilitation program following a traumatic SCI caused by a mechanical fall. At admission, the International Standard for Neurological Classification of SCI (ISNCSCI) exam demonstrated R C3 L C4 sensory, R C3 L C5 motor, and American Spinal Injury Association (ASIA) impairment score (AIS) C tetraplegia. One week after admission to inpatient rehabilitation, the patient developed right lower extremity swelling with no edema. This was following recent surgery of cervical spine fusion, while on prophylactic dosing of anticoagulation of enoxaparin 40 milligrams (mg) daily. Concern for DVT prompted a diagnostic lower extremity ultrasound examination. Ultrasound exam revealed thrombosis in the right posterior tibial and right peroneal veins. Notably, this was a targeted ultrasound for symptomatic presentation, not a routine screening. At the time of the below the right knee DVT diagnosis, the patient was three weeks post-operation and was not cleared for full dose anticoagulation until four weeks post-operation, per the spine surgeon. Thus, the patient was offered an inferior vena cava (IVC) filter versus repeat ultrasound in one week to monitor below the knee DVT. The patient did not agree to IVC filter and rather agreed to have a repeat ultrasound in one week.

One week after the DVT diagnosis, the patient received a repeat ultrasound prior to initiating a full anticoagulation regimen. At this time, the patient was four weeks post-operation and cleared by spine surgeon to initiate full dose anticoagulation. The scan showed no changes in the below-the-knee DVT, and the patient began apixaban 10 mg twice daily for one week then 5 mg twice daily. Two weeks later, he developed perirectal bleeding. The patient remained hemodynamically stable throughout this episode. However, his hemoglobin levels decreased from 12.4 grams per deciliter (gm/dL) to 10.2 gm/dL. D-dimer testing was not performed for the lower extremity swelling, as ultrasound was readily available. Although the patient had surpassed the four-week post-operation mark when the perirectal bleeding occurred, this complication demonstrated that full anticoagulation remained contraindicated. As a result, the decision was made to place an IVC filter at this time.

The bleeding resolved after two weeks. At that time, another diagnostic ultrasound examination was ordered to evaluate the status of the right lower extremity DVT. The ultrasound examination showed progression of the right lower extremity DVT proximally, as well as a new DVT seen in the left lower extremity, noting DVTs in the bilateral external iliac veins through the posterior tibial veins. Anticoagulation was then restarted with enoxaparin 1 mg per kilogram (kg) every 12 h. Four weeks later, the patient was diagnosed with Clostridium Difficile (*C. difficile*). This further exacerbated his UC, with new presentation of hematochezia despite being treated with mesalamine and prednisone for his UC. Anticoagulation with enoxaparin was discontinued again, and he received a vedolizumab infusion and a prednisone taper to address the UC exacerbation in the setting of the active *C. difficile* infection.

A full anticoagulation regimen with enoxaparin 1 mg per kg every 12 h was resumed three weeks later when the patient was negative for occult blood for 14 days. Throughout this course, the patient remained hemodynamically stable and, while on enteric precautions, continued bed-level rehab therapies as tolerated.

## Discussion

This case highlights the challenges in managing DVT in post-surgical patients, specifically in patients with tetraplegia and bleeding risks. Acute trauma and chronic inflammation significantly contribute to a prothrombotic state, substantially increasing a patient’s risk of experiencing a VTE event [3, 4]. The heightened risk is due to the complex interplay between coagulation activation and inflammatory responses following injury, which can lead to endothelial dysfunction, platelet activation, and alterations in coagulation system [3].

The incidence of DVT in patients with SCI varies widely depending on the study and whether prophylaxis is used. Without prophylaxis, the incidence can be as high as 47–100% [5]. With prophylaxis, the incidence ranges from 5.3–64% [5]. A recent meta-analysis found the overall incidence of DVT after SCI to be 9.3% (95% confidence interval (CI):8.2%–10.6%) [6].

Risk factors for DVT in SCI patients include:

1. Degree of paralysis: Complete paralysis is associated with a higher risk (odds ratio (OR) = 3.69, 95% CI: 2.60, 5.24) [7].
2. Age: Middle-aged and older patients have a higher risk (OR = 2.08, 95% CI: 1.47, 2.95) [7].
3. Time since injury: DVT risk is highest in the acute phase, with peak incidence between 7 and 10 days post-injury [8].
4. Level of injury: Paraplegia is associated with increased risk (OR = 1.81, 95% CI: 1.49, 2.19) [7].
5. Other factors: Male sex, personal/family history of venous thrombosis, smoking history, lack of compression therapy, presence of lower limb/pelvic fracture, and diabetes are all associated with increased DVT risk in SCI patients [7].

Regarding risk factors for the development of DVT in flaccid versus spastic paralysis, one study noted that among patients who developed DVT, some had no spasticity while others had grade 1 spasticity, suggesting that both flaccid and spastic conditions may be associated with DVT risk [8].

Moreover, serial ultrasounds have demonstrated that below-the-knee DVT can progress in both SCI and non-SCI trauma patients, as was observed in this case [9, 10]. The progression of distal DVT to proximal veins occurs in approximately 8–15% of untreated patients, highlighting the importance of vigilant monitoring and appropriate intervention [11]. Inflammatory bowel disease (IBD) poses a particular concern in SCI patients, as the risk of developing VTE is 2-to-3 times higher in these patients compared to the general population [2, 12, 13]. The elevated risk is more pronounced than in other autoimmune inflammatory disease such as rheumatoid arthritis or celiac disease [13]. As such, special considerations in patient management need to be made when patients present with a combination of IBD, particularly UC, and an acute traumatic injury like SCI (Tables 1, 2).

**Table 1:** SCI bowel program considerations in setting of ulcerative colitis flare.

**SCI impact on UC course**
Neurogenic obesity results in increased adipose, which in turn releases pro-inflammatory adipokines, resulting in low grade systemic inflammation [17]
**Considerations**
Hold bowel program during UC flare
Resume bowel program as soon as clinical UC remission
May increase likelihood of colostomy long term [17]
Obtain CBC, C-reactive protein, and fecal calprotectin
Consult gastroenterologist to optimize therapy and consider colonoscopy
**Avoid during UC flare**
Manual dis-impaction
Transanal irrigation during [17]
Dietary fiber [18]

*CBC* complete blood count, *UC* ulcerative colitis, *SCI* spinal cord injury.

**Table 2:** Thromboembolism in the setting of SCI vs SCI and ulcerative colitis.

**Prophylaxis considerations in SCI**
Highest risk during acute-care phase, within first 2 weeks of injury [19]
Anticoagulation thromboprophylaxis for first 8 weeks after injury [19]
Acute phase: LMWH is preferred [19]
Post-acute and rehabilitation phase: Use LMWH, DOAC, or warfarin [19]
Note: IVC filter is not a first line prophylaxis [19]
**DVT treatment considerations**
Below the knee DVT
Full dose anticoagulation vs serial ultrasounds, or filter if anticoagulation is contraindicated
**Above the knee DVT**
Full dose anticoagulation, or filter if anticoagulation is contraindicated
**Considerations in SCI and ulcerative colitis**
Increased risk of VTE during active UC flare
Relative risk higher during non-hospitalized periods vs hospitalized periods [20]
Monitor for frank blood, occult blood, and trend hemoglobin and hematocrit
Hold anticoagulation if down trending of hemoglobin/hematocrit, or bleeding
Consider monitoring with bilateral lower extremity doppler
Depending on severity of DVT and response to anticoagulation, consider IVC filter vs thrombectomy
Consider delay in corticosteroids for UC remission, due to increased risk of VTE [2]
Consider VTE prophylaxis for all future hospitalizations

*DOAC* direct oral anticoagulant, *DVT* deep venous thrombus, *IVC Filter* inferior vena cava filter, *LMWH* low molecular weight heparin, *SCI* spinal cord injury, *UC* ulcerative colitis, *VTE* venous thromboembolism.

Furthermore, though D-dimers have the potential to be a screening tool for DVT, D-dimer elevation is commonly seen after recent surgery [14, 15]. For this case the patient was not cleared for full dose anticoagulation until four weeks post-operation. The right lower extremity DVT occurred within this timeframe, precluding the initiation of full dose anticoagulation therapy. Although the patient had surpassed the four-week post-operation mark when the perirectal bleeding occurred, this complication demonstrated that full anticoagulation remained contraindicated and the treatment strategy modified.

Additionally, the patient’s treatment was further complicated due to a previous history of recurrent urinary tract infections and antibiotic use. This clinical background significantly increased the risk of *C. difficile* infection in the context of UC, where luminal gut microbiota is already altered and intestinal permeability is enhanced [16]. Thus, the bowel management program required careful adjustments to address the UC exacerbation, which was likely further triggered by *C. difficile* infection. Given the heightened risk in VTE in IBD patients, particularly during disease flares, studies suggest that patients with IBD, such as UC, would benefit from VTE prophylaxis post-discharge from hospitalization [5, 13]. This approach could potentially mitigate the risk of thrombotic events in this high-risk population.

The management of patients with concurrent IBD and acute traumatic injuries requires a multifaceted approach that addresses both the immediate trauma-related concerns and the chronic inflammatory condition. This case report underscores the importance of recognizing the additive prothrombotic effects of UC and tetraplegia. It highlights the intricate interplay in DVT management in the context of lower GI bleeding. Future studies are needed to explore the risks and benefits of continuing biologic agents, such as adalimumab, and other UC remission maintenance therapies in the acute stages of traumatic SCI to limit UC exacerbations and decrease an already hypercoagulable state.

In conclusion, this complex case of the triad of recent SCI, acute DVT – initially below and then above the knee – and UC with its prothrombotic effects illustrates that the optimal approach may be in favor of early treatment and increased surveillance given the compounded risks they present. Careful consideration of VTE prophylaxis, bowel management, and infection control is crucial for optimizing patient outcomes in these complex cases.

### Reporting summary

Further information on research design is available in the Nature Research Reporting Summary linked to this article.

## Supplementary information

CARE Checklist

Reporting Summary

Consent

## Data Availability

Data was gathered at Gaylord Hospital, part of Gaylord Specialty Healthcare. Additionally, data is available from the corresponding author on reasonable request.
