# Supplementary material for: Multifaceted challenges of deep venous thrombosis in the setting tetraplegia and ulcerative colitis: case report
Source: Spinal Cord Ser Cases. 2025 Apr 1;11:8. doi: 10.1038/s41394-025-00703-3 (PMC11961597; doi:10.1038/s41394-025-00703-3)
Supplement: Supplementary file 1 — CARE Checklist [file 41394_2025_703_MOESM1_ESM.pdf]

CARE
case report guidelines

CARE Checklist of information to include when writing a case report

(cc) BY-NC-ND

Topic	Item	Checklist item description	Reported on Line
**Title**	1	The diagnosis or intervention of primary focus followed by the words "case report"	1-2
**Key Words**	2	2 to 5 key words that identify diagnoses or interventions in this case report, including "case report"	35-36
**Abstract (no references)**	3a	Introduction: What is unique about this case and what does it add to the scientific literature?	16-20
	3b	Main symptoms and/or important clinical findings	21-28
	3c	The main diagnoses, therapeutic interventions, and outcomes	21-28
	3d	Conclusion—What is the main "take-away" lesson(s) from this case?	29-33
**Introduction**	4	One or two paragraphs summarizing why this case is unique (**may include references**)	38-50
**Patient Information**	5a	De-identified patient specific information.	53-55
	5b	Primary concerns and symptoms of the patient.	58-60
	5c	Medical, family, and psycho-social history including relevant genetic information	53-57
	5d	Relevant past interventions with outcomes	59-60
**Clinical Findings**	6	Describe significant physical examination (PE) and important clinical findings.	59-62
**Timeline**	7	Historical and current information from this episode of care organized as a timeline	53-91
**Diagnostic Assessment**	8a	Diagnostic testing (such as PE, laboratory testing, imaging, surveys).	60-62, 68-72, 78-83, 84-91
	8b	Diagnostic challenges (such as access to testing, financial, or cultural)	n/a
	8c	Diagnosis (including other diagnoses considered)	61-63, 83-87
	8d	Prognosis (such as staging in oncology) where applicable	n/a
**Therapeutic Intervention**	9a	Types of therapeutic intervention (such as pharmacologic, surgical, preventive, self-care)	59-60, 71, 77, 85-89
	9b	Administration of therapeutic intervention (such as dosage, strength, duration)	59-60, 71-73, 76-77, 82-83, 84-89
	9c	Changes in therapeutic intervention (with rationale)	62-89
**Follow-up and Outcomes**	10a	Clinician and patient-assessed outcomes (if available)	88-91
	10b	Important follow-up diagnostic and other test results	68-70, 78-82,84-87
	10c	Intervention adherence and tolerability (How was this assessed?)	88-91
	10d	Adverse and unanticipated events	72-87
**Discussion**	11a	A scientific discussion of the strengths AND limitations associated with this case report	100-137
	11b	Discussion of the relevant medical literature **with references**.	93-158
	11c	The scientific rationale for any conclusions (including assessment of possible causes)	93-158
	11d	The primary "take-away" lessons of this case report (without references) in a one paragraph conclusion	154-158
**Patient Perspective**	12	The patient should share their perspective in one to two paragraphs on the treatment(s) they received	n/a
**Informed Consent**	13	Did the patient give informed consent? Please provide if requested	Yes ☑ No ☐
