# Supplementary material for: Multifaceted challenges of deep venous thrombosis in the setting tetraplegia and ulcerative colitis: case report
Source: Spinal Cord Ser Cases. 2025 Apr 1;11:8. doi: 10.1038/s41394-025-00703-3 (PMC11961597; doi:10.1038/s41394-025-00703-3)
Supplement: Supplementary file 3 — Consent [file 41394_2025_703_MOESM3_ESM.pdf]

Gaylord Hospital, Inc.

Consent Form for Case Reports

Working Title:

Principal Investigator Seeking Consent:

You are being asked to consider allowing Dr Rosenblum and researchers they are working with, to use information about your medical history and rehabilitation to write what is called a case report. Case reports are typically used to share new unique information experienced by one patient during their clinical care that may be useful for other physicians and members of a health care team. A case report may be published in a variety of formats. This may include in print and/or shared on the internet for others to read, and/or presented at professional conferences. This form explains the purpose of this case report. Please read this form carefully and take your time to make your decision. You should ask any questions that you may have or ask to have the entire process described to you.

The purpose of this case report is to Describe ulcerative colitis + SCI risks + blood clot

Your information being used for this case report may include medical information from your acute care stay, other rehabilitation hospital, and medical information from your stay at Gaylord Hospital.

Dr Rosenblum, at Gaylord Hospital, is obligated to protect your privacy and not disclose your personal information (name, date of birth, and medical record number). When the case report is published or presented, your identity will not be disclosed.

Although your personal information collected or obtained will be kept confidential and protected to the fullest extent of the law, there is a limited risk associated with this case report that could result in a loss of confidentiality by virtue of your unique experience.

It is not expected that you will directly benefit from participating in this case report. The information that can be shared with other health care professionals, however, may improve the care that is received by you and others in the future.

Allowing your information to be used in this case report will not involve any additional costs to you. You will not receive any compensation.

Taking part in this case report is your choice. You may choose not to take part or you may change your mind at any time. However, once the case report is written and published, it will not be possible for you to withdraw it. Your decision will not result in any penalty or loss of benefits to which you are entitled including the quality of care you receive.

You will be told about any new information relating to this case report that may affect you.

Your signature below means that you have read the above information about this Case Report and have had a chance to ask questions to help you understand how your information will be used and that you give permission to allow your information to be used in this case report.

If you have any questions please contact Dr Rosenblum.

Form #: 9120-300
Approved Date: 11/15/2021

Gaylord Hospital, Inc.

CASE STUDY - CONSENT TO PARTICIPATE

Name of Participant: [redacted] Date of Birth [redacted]

Participant/Legally Authorized Representative

By signing this form, I confirm that:

- The case report has been fully explained to me and all of my questions have been answered to my satisfaction
- I have been informed of the risks and benefits, if any, of allowing my information to be used in this case report
- I have been informed that I do not have to participate in this case report
- I have read each page of this form
- I authorize access to my personal health information (medical record) as explained in this form
- I have agreed to participate in this case report

witnessed by Dr. Priscilla Mapelli, DO 11/8/23

[redacted] Pt unable to sign 11/8/2023

Name [redacted] Authorized Representative (print) | Signature | Date

witnessed by significant other [redacted] by phone and Dr Mapelli

I have carefully explained to the subject the nature of the above project. I hereby certify that to the best of my knowledge the person who is signing this consent form understands clearly the nature involved in their participation, and their signature is legally valid. A medical problem or language or educational barrier has not precluded this understanding.

David [illegible] 11/8/2023

Name of Person obtaining Consent Authorized Representative (print) | Signature | Date

Form #: 9120-300
Approved Date: 11/15/2021
